# LncRNA RP1-85F18.6 affects osteoblast cells by regulating the cell cycle

**DOI:** 10.1515/biol-2020-0090

**Published:** 2020-12-22

**Authors:** Jiangtao Song, Wenrong Song, Lei Zhang

**Affiliations:** Orthopedics Department, Hanchuan People’s Hospital, Hanchuan City, Hubei Province 431600, China; Department of Endocrinology, Hanchuan People’s Hospital, Hanchuan City, Hubei Province 431600, China

**Keywords:** lncRNA, RP1-85F18.6, cell cycle, osteoblast

## Abstract

A lncRNA RP1-85F18.6 was reported to affect cell growth by regulating the cell cycle. Here we tested whether it affects the proliferation of osteoblast cells by regulating the cell cycle. We determined the expression of RP1-85F18.6 in two osteoblast cell lines hFOB and HOB by qPCR. Then we knocked down or overexpressed RP1-85F18.6 in hFOB and tested the alteration of viability, cell cycle, and cell cycle regulatory proteins. Results showed that both hFOB and HOB expressed RP1-85F18.6. The knockdown of RP1-85F18.6 decreased the viability of hFOB, while the overexpression of it increased the viability. Higher expression of RP1-85F18.6 results in higher cell viability. The knockdown of RP1-85F18.6 caused an increase in the S phase cells and a decrease in the G2/M phase cells. The overexpression of RP1-85F18.6 caused a decrease in the S phase cells and an increase in the G2/M phase cells. The knockdown of RP1-85F18.6 decreased cyclin A, cdk1, E2F, cyclin B, p53, and p21, whereas the overexpression of RP1-85F18.6 increased cyclin A, cdk1, E2F, cyclin B, p53, and p21. This study demonstrated that RP1-85F18.6 is expressed in osteoblast cell lines hFOB and HOB. RP1-85F18.6 affects the proliferation of osteoblasts by regulating the cell cycle.

## 1 Introduction

Osteoporosis, characterized by significant bone mineral density decrease, is a common metabolic bone disease, which results in fragility fractures or even a fatal outcome. Annually, about 9,000,000 fracture cases are caused by osteoporosis worldwide [1]. Approximately, 30% of women in the United States develop postmenopausal osteoporosis [2], which is the most common bone disease in elderly women [3]. So far, many studies have revealed the pathological mechanisms [4] and developed therapeutics [5,6] for this disease, but more effort is required to further understand osteoporosis.

Long non-coding RNAs (lncRNAs) are RNA transcripts with lengths over 200 nucleotides but are not translated into proteins [7]. Much as the exact functions of lncRNAs remain unclear, they are involved in transcriptional regulation of many genes [8]. The expression of certain non-coding RNAs was reported to impact the bone tissue [9]. Many lncRNAs were also shown to be associated with some human diseases [10,11,12]. Recently, the expression of lncRNA in bone was recognized as a potential osteoporosis risk factor for premenopausal women [13]. Certain lncRNAs were proved to affect bone cells, including osteoblasts [14,15,16]. As the key cells for bone formation, osteoblast cells can differentiate and further form mature bone tissue [17]. Studies revealed that the lncRNAs expressed in bone play a critical role in regulating bone formation [18]. LncRNA H19 was reported to promote differentiation of osteoblast cells [19,20,21]. However, the effect of lncRNAs on the growth of osteoblasts has not been fully elucidated.

A newly identified lncRNA, RP1-85F18.6, which is located on human chromosome 22 [22], was found to affect cell growth by regulating the cell cycle [23]. Here we hypothesized that RP1-85F18.6 affects the proliferation of osteoblasts by regulating the cell cycle. In this study, we tested the expression of RP1-85F18.6 in osteoblast cell lines and determined its effect on the cell cycle. Our study contributed to the development of RP1-85F18.6 as a novel target for the treatment and diagnosis of osteoporosis.

## 2 Materials and methods

### 2.1 Cell lines and cell culture

Human colon cancer cell line SW620 [SW-620] (ATCC^®^ CCL-227) and human osteoblast cell line hFOB 1.19 (ATCC^®^ CRL-11372™) were purchased from ATCC (Washington, USA). The primary human osteoblast (HOB) cell line was purchased from PromoCell (Heidelberg, Germany). SW620 was cultured in Dulbecco’s modified Eagle’s medium containing 10% fetal bovine serum, and the other two cell lines were cultured in Osteoblast Growth Medium C-27001 (Heidelberg, Germany) in a humidified atmosphere of 5% CO_2_ at 37°C.

### 2.2 MTT

Cell viability was determined by an MTT assay, which was described previously [24]. The cells were cultured in 96-well plates. Then, 20 µL of 5 mg/mL MTT solution (Abcam, Cambridge, UK) was added to the cells. After 2 h, dimethyl sulfoxide (200 µL) was added to the wells. Absorbance was measured at 490 nm using a microplate reader. The experiment was repeated three times, and all the data were normalized and plotted.

### 2.3 RT-qPCR

The expression of RP1-85F18.6 was determined using a qPCR assay, according to the method that was described previously [25]. TRIzol reagent (Thermo Fisher Scientific, Inc.) was used to isolate total RNA from the cells. Then, the total RNA was reverse-transcribed into cDNA using a reverse transcription kit (Thermo, USA). Quantitative real time-PCR was performed with a PowerUp™ SYBR™ Green Master Mix (Thermo, USA) using a Roche LightCycler 480 Sequence Detection System. The protocol for PCR is as follows: 95°C for 3 min, 40 cycles of 95°C for 30 s, 58°C for 15 s, and 72°C for 30 s. Gene expression was quantified using the 2^−ΔΔCT^ method.

The primers used for RT-qPCR were obtained from Sigma-Aldrich, Inc. (USA), and the sequences are as follows: GAPDH forward, 5′-GAAGGTGAAGGTCGGAGTC-3′; GAPDH reverse, 5′-GAAGATGGTGATGGGATTTC-3′. LncRNA RP1-85F18.6 forward, 5′-GGCTCTTTGCTCACATCG-3′; reverse, 5′-AAGGAAACCACAGGCTCA-3′.

### 2.4 Cell transfection

The knockdown and overexpression of RP1-85F18.6 were achieved by the transfection of the siRNA or expression vector. The cells were transfected using Lipofectamine^®^ 2000 (Invitrogen; Thermo Fisher Scientific, Inc.), which was described previously [26].

RP1-85F18.6 knockdown: lncRNA RP1-85F18.6 small interfering (si)RNA and negative control (NC) siRNA were purchased from Guangzhou RiboBio Company (Guangzhou, China). The sequences were as follows: lncRNA RP1-85F18.6 siRNA, 5′-GACTCCGCCGTGAACCCTTCA-3′. A scramble siRNA (siN05815122147) was used as the NC siRNA. The transfection concentration of siRNA was 60 nM.

RP1-85F18.6 overexpression: the entire sequence of human lncRNA RP1-85F18.6 was amplified from hFOB cell lines using PCR and cloned into the pcDNA3.1 vector. The NC empty vector, which was purchased from Shanghai GeneChem Co., Ltd (Shanghai, China), and the lncRNA RP1-85F18.6 plasmid were transfected into hFOB using Lipofectamine^®^ 2000. The transfection concentration of the plasmid was 2 µg/mL.

### 2.5 Cell cycle flow cytometry

The cell cycle was analyzed using flow cytometry with propidium iodide (PI) staining, which was described previously [27]. Briefly, cells were washed with PBS and resuspended at a concentration of 1  ×  10^6^/mL. Then, the cells were fixed with 100% ethanol and incubated for 3 h at 4°C. Then, the suspended cells were washed with PBS twice and added with PI staining solution (0.1% Triton X-100 and 0.2 mg/mL DNAse-free RNAse A, 0.02 mg/mL in cold PBS). Then, the cells were incubated at 37°C for 15 min for staining. A BD FACSCalibur was used to acquire cell cycle data. FlowJo Version 10 was used for the cell cycle analysis.

### 2.6 Western blotting

Protein expression was determined by a western blotting assay, which was described previously [28]. Proteins were extracted from cells using RIPA buffer with a protease inhibitor (Sigma-Aldrich, USA). Total protein concentrations were determined using a BCA protein assay kit to control the loading amount in each well (30 μg). SDS gel electrophoresis was performed to separate proteins, and the proteins were transferred onto 0.2 μm polyvinylidene difluoride membranes, which were subsequently blocked with 5% skimmed milk in Tris-buffered saline with 0.5% Tween-20 (TBST). The membranes were then reacted with primary (1:1,000 dilution of BSA) and secondary antibodies (1:3,000 dilution of BSA) subsequently. The band intensities were photographed using a gel imaging system after reaction with ECL reagents. The images were quantified using Quantity One^®^ analysis software version 4.3.0. (Bio-Rad, Hercules, CA, USA). The experiment was repeated more than three times.

The primary antibodies used in the experiment are as follows: anti-cyclin A antibody (sc-271682), anti-cdk1 antibody (ab18), anti-E2F antibody (ab179445), anti-cyclin B antibody (ab72), anti-p53 antibody (ab26), anti-p21 antibody (ab109520), cyclin D1 antibody (#2922), anti-Cdk2 antibody (ab32147), anti-cyclin E1 antibody (ab33911), and anti-Cdk2 antibody (ab32147). The secondary antibodies were all purchased from Sigma-Aldrich (USA).

### 2.7 Statistical analysis

All the experiments were repeated three times. *T*-test or one-way ANOVA and Dunnett’s *post hoc* tests were used to compare the significant difference between the control and experimental groups (*p*  <  0.05). GraphPad Prism (version 6) was used to plot the figures and calculate statistics.

## 3 Results

### 3.1 LncRNA RP1-85F18.6 was expressed in hFOB and HOB

**Figure 1:**
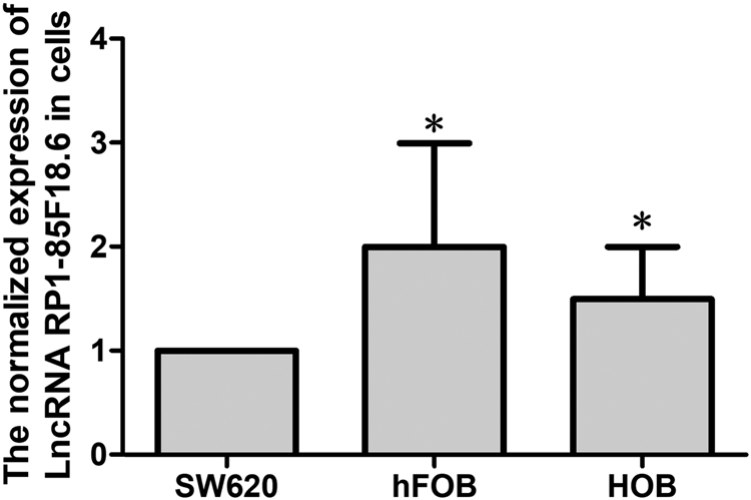
The expression of lncRNA RP1-85F18.6 in osteoblast cell lines (*p  <  0.01). Colon cancer cell line SW620 was used as a positive control.

Here we tested the expression of lncRNA RP1-85F18.6 in two osteoblast cell lines hFOB and HOB by a PCR assay. Colon cancer cell line SW620, which was reported to have RP1-85F18.6 expression, was used as the positive control [29]. Results showed that lncRNA RP1-85F18.6 was expressed in both cell lines (Figure 1).

### 3.2 The knockdown and overexpression of RP1-85F18.6 in hFOB

**Figure 2:**
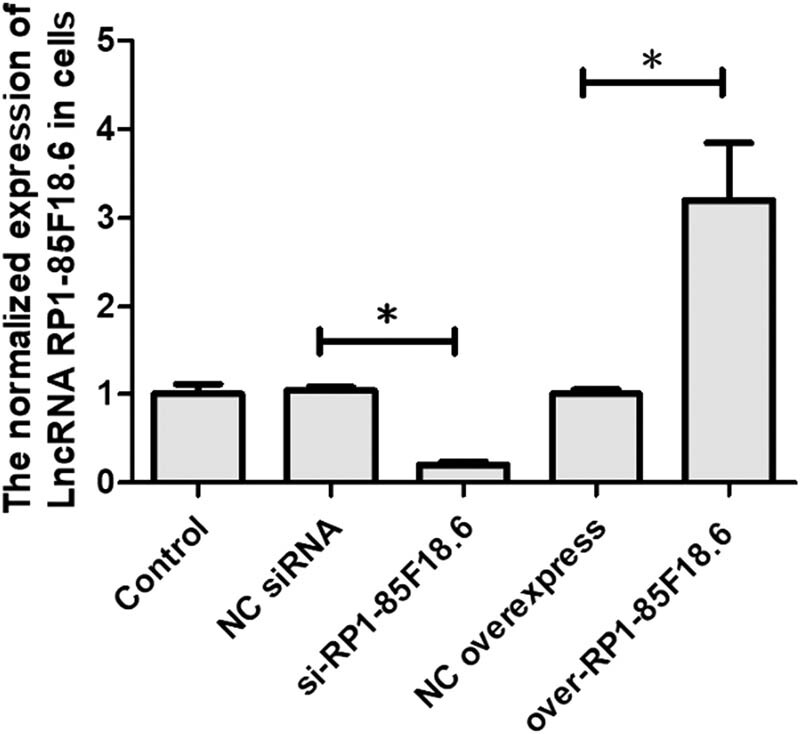
The knockdown and overexpression of RP1-85F18.6 (*p  <  0.01).

To further explore the role of RP1-85F18.6 in osteoblasts, we selected one of the osteoblast cell lines hFOB, which has a higher expression of RP1-85F18.6, to conduct the subsequent study. We knocked down or overexpressed RP1-85F18.6 in hFOB and confirmed the expression by a PCR assay. Results showed that compared to blank control (control), the expressions of RP1-85F18.6 in NC for silencing RNA (NC siRNA) and NC for overexpression (NC overexpress) were not significantly different, while the expression in RP1-85F18.6 knockdown cells (si-RP1-85F18.6) was significantly decreased, and the expression in RP1-85F18.6 overexpressing cells (over-RP1-85F18.6) was significantly increased. This indicated that both the knockdown and overexpression were successful (Figure 2).

### 3.3 LncRNA RP1-85F18.6 affected cell viability of hFOB

**Figure 3:**
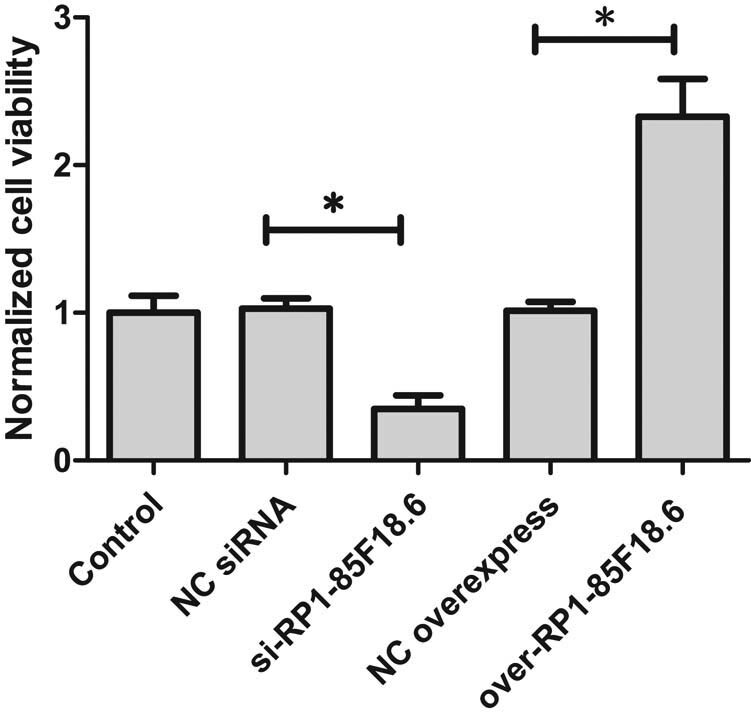
Effect of the expression of RP1-85F18.6 on the viability of osteoblasts (*p  <  0.01).

To test whether RP1-85F18.6 affects cell proliferation, we compared the viability of knocked-down cells, overexpressing cells, and wild-type osteoblasts using an MTT assay. MTT assay has been widely used in cell proliferation study [37]. Results showed that, compared to the control, the expressions of RP1-85F18.6 in NC siRNA and NC overexpress were not significantly different, indicating that the transfection had no effect on cell viability. The knockdown of RP1-85F18.6 significantly decreased cell viability and the overexpression of RP1-85F18.6 significantly increased cell viability (Figure 3). These results indicated that the expression of RP1-85F18.6 was associated with cell viability.

### 3.4 LncRNA RP1-85F18.6 affected the cell cycle of hFOB

**Figure 4:**
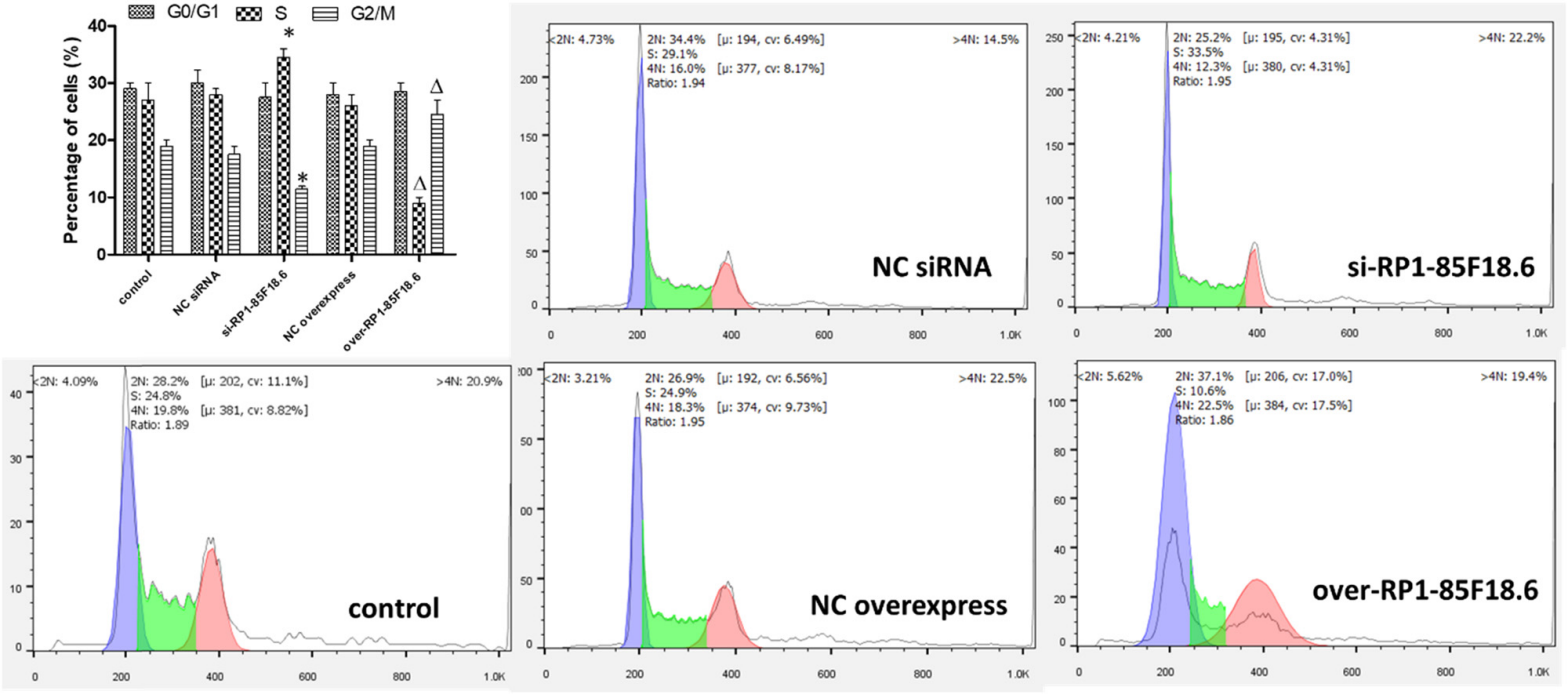
The cell cycle of cells was determined by a flow cytometry assay with PI staining (significant differences from NC siRNA and NC overexpress are indicated by *p  <  0.05 and ^Δ^ *p*  <  0.05, respectively).

To further explore the effect of RP1-85F18.6 on cell growth of osteoblasts, we analyzed the cell cycle of hFOB cells using a flow cytometry assay with PI staining. Results showed that compared to the control, the cell cycles of NC siRNA and NC overexpress were not significantly different, indicating that the transfection had no effect on the cell cycle. The knockdown of RP1-85F18.6 had no effect on the G0/G1 phase cells but caused a significant increase in the S phase cells and a decrease in the G2/M phase cells. On the other hand, the overexpression of RP1-85F18.6 also had no significant effect on the G0/G1 phase cells but caused a decrease in the S phase cells and an increase in the G2/M phase cells. This indicated that the expression of RP1-85F18.6 affected osteoblast cells in the S and G2/M phase (Figure 4). Therefore, we suggested that RP1-85F18.6 promotes cell growth by regulating the cell cycle.

### 3.5 LncRNA RP1-85F18.6 affected the expression of cell cycle-related proteins in hFOB

**Figure 5:**
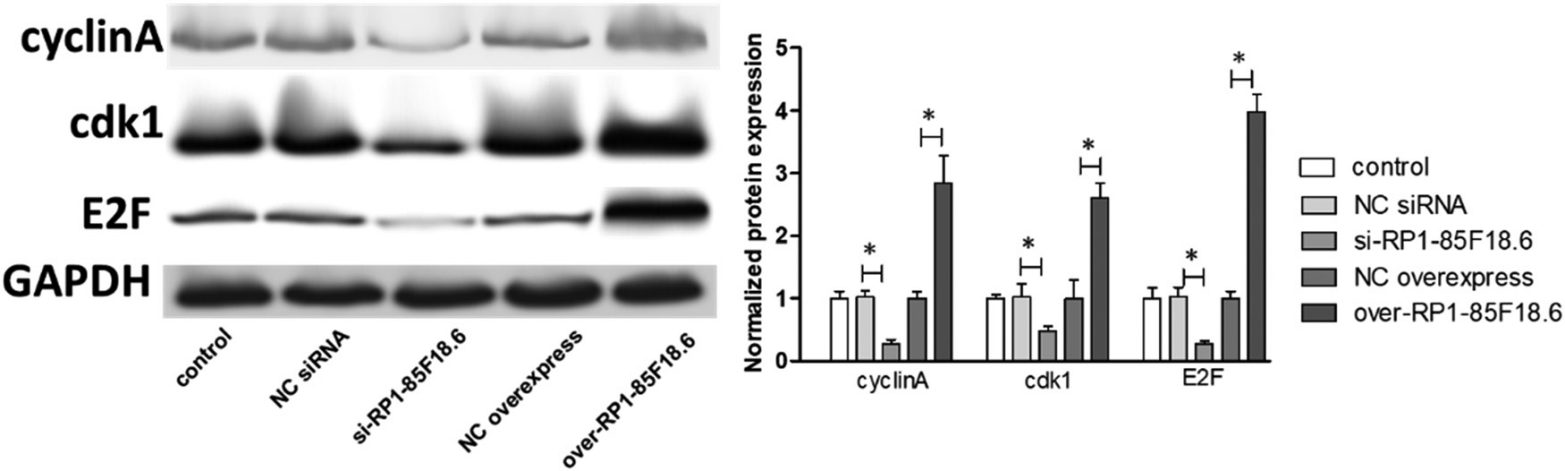
Effect of lncRNA RP1-85F18.6 on the expression of cyclin A related proteins (significant differences are indicated by *p  <  0.01).

**Figure 6:**
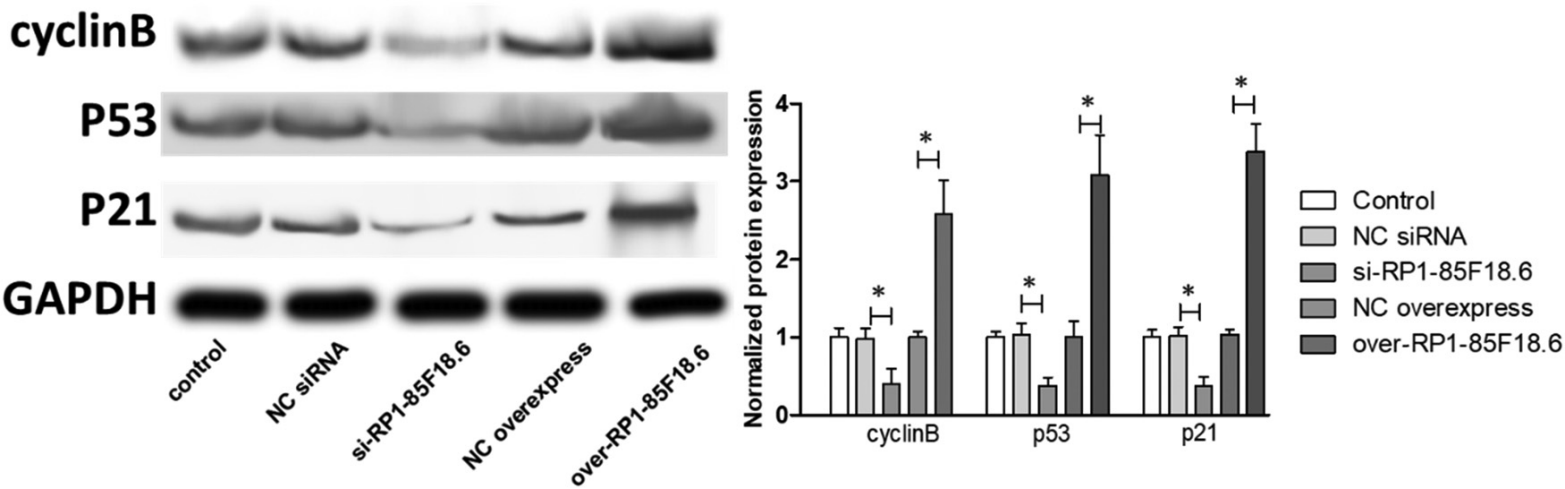
Effect of lncRNA RP1-85F18.6 on the expression of cyclin B related proteins (*p  <  0.01).

**Figure 7:**
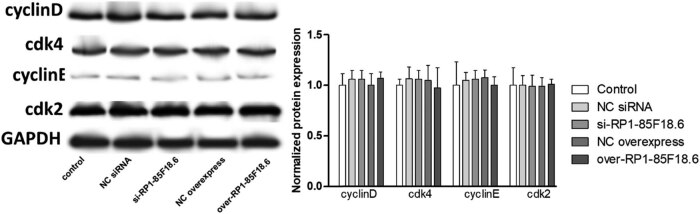
Effect of lncRNA RP1-85F18.6 on the expression of cyclin D and cyclin E related proteins (*p  <  0.05).

**Figure 8:**
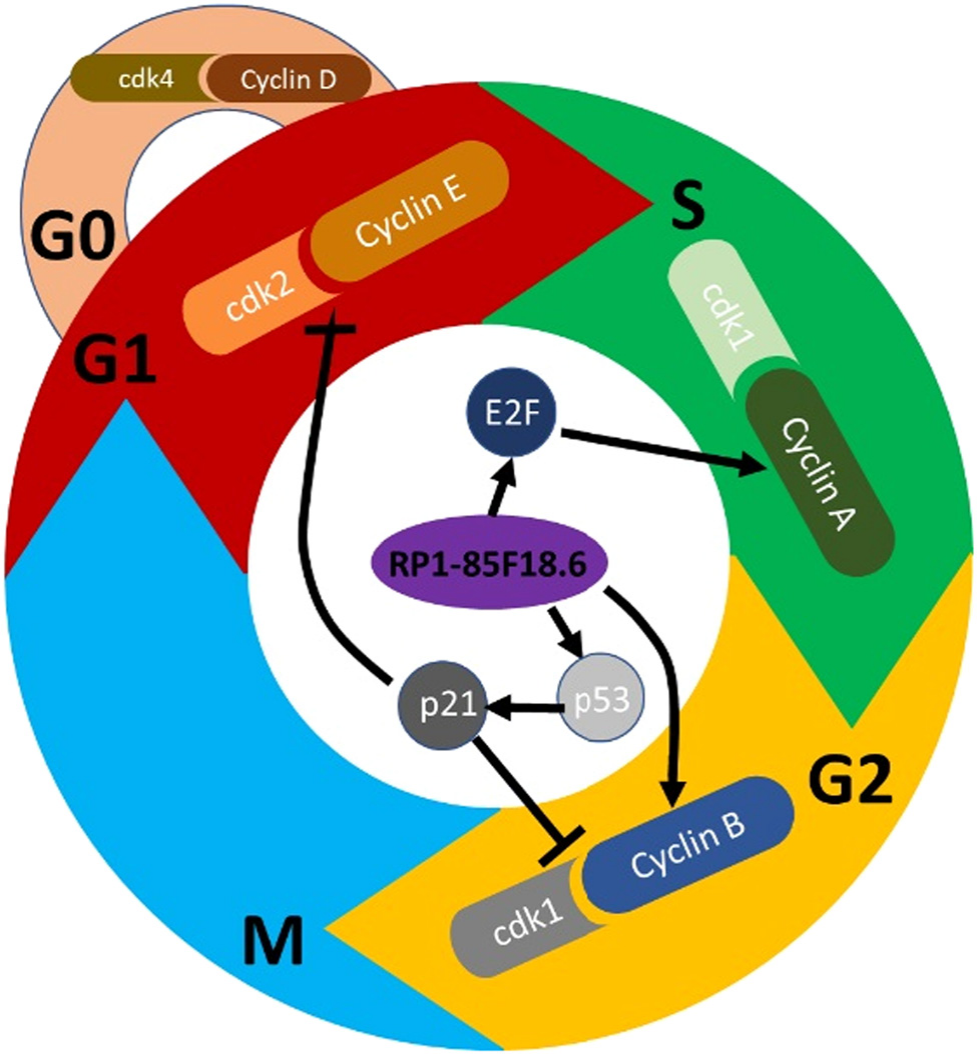
Mechanism of the effect of RP1-85F18.6 on the cell cycle.

To identify the potential targets involved in the effect of RP1-85F18.6 on the cell cycle, we determined several cell cycle-related proteins by using western blotting. First, we determined the expression of cyclin A, cdk1, and E2F proteins. Cyclin A and cdk1 are responsible for S phase regulation [30]. Results showed that the transfection had no effect on the expression of all the proteins studied, whereas the knockdown of RP1-85F18.6 decreased the expression of cyclin A, cdk1, and E2F. The overexpression of RP1-85F18.6 did the opposite: it increased the expression of cyclin A, cdk1, and E2F (Figure 5). Then, we determined the expression of cyclin B, p53, and p21 proteins. The p53/21 pathway is responsible for the negative regulation of cyclin B and cdk1, regulating activities of the G2 phase [31]. Results showed that the transfection had no effect on the expression of all the proteins studied (all *p* > 0.05). The knockdown of RP1-85F18.6 decreased the expression of cyclin B, p53, and p21 (all *p* < 0.01), whereas the overexpression of RP1-85F18.6 increased the expression of cyclin B, p53, and p21 (all *p* < 0.01) (Figure 6). Moreover, we also determined the expression of cyclin D, cdk4, cyclin E, and cdk2 proteins. Cyclin D and cyclin E are the regulators for cell activities in the G1 phase [32]. Results showed that the expressions of cyclin D, cdk4, cyclin E, and cdk2 in control, NC siRNA, si-RP1-85F18.6, NC overexpress, and over-RP1-85F18.6 groups were not significantly different (Figure 7). Therefore, RP1-85F18.6 might promote cell growth by regulating cyclin proteins. We proposed a mechanism for the regulation of RP1-85F18.6, which is discussed in the Discussion section (Figure 8).

## 4 Discussion

This study tried to reveal the role of a newly discovered lncRNA RP1-85F18.6 in the proliferation of osteoblasts. We confirmed that RP1-85F18.6 was expressed in both adult (HOB) and fetus (hFOB) derived osteoblast cell lines. These two cell lines are different in their capability of proliferation and differentiation, but it is unknown whether this is associated with their difference in RP1-85F18.6 expression levels. In this study, we focused only on one of these cell lines, hFOB. The reason for selecting hFOB is that hFOB has higher expression of RP1-85F18.6, and it is a stable cell line for transfection [33]. Previously, RP1-85F18.6 was reported to be knocked down and overexpressed in cells successfully [34]. In our study, the expression of RP1-85F18.6 was closely related to the cell viability of hFOB, suggesting that RP1-85F18.6 might promote cell growth.

RP1-85F18.6 was reported to increase colon cancer cell growth [34], and we assumed that the osteoblast and colon cancer cells might share a common mechanism. Cell cycle analysis showed that RP1-85F18.6 expression affected cell viability through regulating the S and G2/M phases. The G0/G1 phase of the cell cycle is the period for differentiation of osteoblasts [35]. The S phase entrance is critical for cell proliferation, and the arrest of cells in the S phase can result in the suppression of proliferation [36]. To explore the effect of RP1-85F18.6 on the action of the cell cycle, we selected and determined the expression of several cell cycle regulatory proteins. The cell cycle is regulated by cyclins, cyclin-dependent kinases (CDKs), and some transcription factors. Cyclins/CDK complexes are the most critical regulators for cell cycle regulation [35]. In this study, we determined the expression of four pairs of cyclins/CDK complexes, which regulate physical processes of each phase in cell cycle, and also some of their upstream regulators.

E2F is a protein that can activate genes required for DNA synthesis and S phase progression. The cyclin A and cdk1, downstream targets of E2F, are responsible for S phase regulation [30]. In this study, the knockdown of RP1-85F18.6 decreased the expression of E2F and further resulted in the decrease of cyclin A and cdk1, whereas the overexpression of RP1-85F18.6 increased the expression of E2F, which accounts for the increase of cyclin A and cdk1.

Besides, the p53/21 pathway is responsible for the negative regulation of cyclin B and cdk1, regulating activities of the G2 phase in cancer cells [34]. The accumulation of p53 can increase the expression of its transcriptional target gene p21, which can potentially inactivate the cyclin B/cdk1 complex [31]. Our results showed that the knockdown or overexpression of RP1-85F18.6 affects the expression of p53, and p53 subsequently affected p21, which negatively regulated activities in the G2 phase, but the increase of cyclin B and cdk1 offset the effect of p21 suppression, and thus the knockdown of RP1-85F18.6 can arrest cells in the S phase, and overexpression of RP1-85F18.6 can promote cells entering the G2 phase. Nevertheless, this study did not demonstrate how they regulate the proliferation in detail, but it provides a potential mechanism that can be validated in the future.

Furthermore, cyclin D and cyclin E are the regulators for cell activities in the G1 phase [32]. Our results showed that the expression of cyclin D, cdk4, cyclin E, and cdk2 was not affected by RP1-85F18.6, which was consistent with the results that the number of cells in the G1/G0 phase was not changed. Besides, p21 can also potentially bind and inactivate the cyclin E/CDK2 complex. This might also account for the change in the number of cells entering the S phase from the G1 phase. However, it is still not clear why the increase of p21 failed to affect cell proliferation, and we supposed a feedback regulation was involved (Figure 8).

In summary, this study demonstrated that lncRNA RP1-85F18.6 plays a role in the proliferation of osteoblasts by affecting their cell cycle by regulating several cell cycle proteins. We suggested that RP1-85F18.6 is an upstream target of cell cycle-related proteins, but whether its regulation is direct or indirect is still unclear and requires more exploration. Additionally, lncRNA H19 was reported to promote osteoblast differentiation [19,20,21]. Further study is needed to explore its effect on the differentiation of osteoblasts. In addition, there might be some linkage between osteoporosis and the decrease of other lncRNAs, which requires more validation. Our study is conducive to the development of RP1-85F18.6 as a novel treatment target for osteoporosis.

## References

[1] Liu GF, Wang ZQ, Liu L, Zhang BT, Miao YY, Yu SN. A network meta-analysis on the short-term efficacy and adverse events of different anti-osteoporosis drugs for the treatment of postmenopausal osteoporosis. J Cell Biochem. 2018;119(6):4469–81. 10.1002/jcb.26550 29227547

[2] Dahl M, Frost L, Søgaard R, Klausen IC, Lorentzen V, Lindholt J. A population-based screening study for cardiovascular diseases and diabetes in Danish postmenopausal women: acceptability and prevalence. BMC Cardiovasc Disord. 2018;18(1):20. 10.1186/s12872-018-0758-8 PMC5800093 29402233

[3] Kahwati LC, Weber RP, Pan H, Gourlay M, LeBlanc E, Coker-Schwimmer M, et al. Vitamin D, calcium, or combined supplementation for the primary prevention of fractures in community-dwelling adults: evidence report and systematic review for the US preventive services task force. JAMA. 2018;319(15):1600–12.10.1001/jama.2017.2164029677308

[4] Liu X, Liu H, Xiong Y, Yang L, Wang C, Zhang R, et al. Postmenopausal osteoporosis is associated with the regulation of SP, CGRP, VIP, and NPY. Biomed Pharmacother. 2018;104:742–50. 10.1016/j.biopha.2018.04.044 29807224

[5] Haixia W, Shu M, Li Y, Panpan W, Kehuan S, Yingquan X, et al. Effectiveness associated with different therapies for senile osteopo-rosis: a network meta-analysis. J Tradit Chin Med. 2020;40(1):17–27. 32227762

[6] Liu H, Xiong Y, Wang H, Yang L, Wang C, Liu X, et al. Effects of water extract from epimedium on neuropeptide signaling in an ovariectomized osteoporosis rat model. J Ethnopharmacol. 2018;221:126–36. 10.1016/j.jep.2018.04.035 29705515

[7] Meseure D, Drak Alsibai K, Nicolas A, Bieche I, Morillon A. Long noncoding RNAs as new architects in cancer epigenetics, prognostic biomarkers, and potential therapeutic targets. Biomed Res Int. 2015;2015(17):320214. 10.1155/2015/320214 PMC4584070 26448935

[8] Ma L, Cao J, Liu L, Du Q, Li Z, Zou D, et al. LncBook: a curated knowledgebase of human long non-coding RNAs. Nucleic Acids Res. 2019;47(D1):D128–34. 10.1093/nar/gky960 PMC6323930 30329098

[9] Li X, Peng B, Zhu X, Wang P, Xiong Y, Liu H, et al. Changes in related circular RNAs following ERbeta knockdown and the relationship to rBMSC osteogenesis. Biochem Biophys Res Commun. 2017;493(1):100–7. 10.1016/j.bbrc.2017.09.068 28919414

[10] Lukiw WJ, Handley P, Wong L, Crapper McLachlan DR. BC200 RNA in normal human neocortex, non-Alzheimer dementia (NAD), and senile dementia of the Alzheimer type (AD). Neurochem Res. 1992;17(6):591–7. 10.1007/BF00968788 1603265

[11] Fu X, Ravindranath L, Tran N, Petrovics G, Srivastava S. Regulation of apoptosis by a prostate-specific and prostate cancer-associated noncoding gene, PCGEM1. DNA Cell Biol. 2006;25(3):135–41. 10.1089/dna.2006.25.135 16569192

[12] Watson JB, Sutcliffe JG. Primate brain-specific cytoplasmic transcript of the Alu repeat family. Mol Cell Biol. 1987;7(9):3324–7. 10.1128/mcb.7.9.3324-3327.1987 PMC367971 2444875

[13] Zhou Y, Xu C, Zhu W, He H, Zhang L, Tang B, et al. Long noncoding RNA analyses for osteoporosis risk in Caucasian women. Calcif Tissue Int. 2019;150(2):183–92. 10.1007/s00223-019-00555-8 PMC6712977 31073748

[14] Ou F, Su K, Sun J, Liao W, Yao Y, Zheng Y, et al. The LncRNA ZBED3-AS1 induces chondrogenesis of human synovial fluid mesenchymal stem cells. Biochem Res Biophys Commun. 2017;487(2):457–63. 10.1016/j.bbrc.2017.04.090 28431932

[15] Zhuang W, Ge X, Yang S, Huang M, Zhuang W, Chen P, et al. Upregulation of lncRNA MEG3 Promotes osteogenic differentiation of mesenchymal stem cells from multiple myeloma patients by targeting BMP4 transcription. Stem Cell. 2015;33(6):1985–97. 10.1002/stem.1989 25753650

[16] Mulati M, Kobayashi Y, Takahashi A, Numata H, Saito M, Hiraoka Y, et al. The long noncoding RNA Crnde regulates osteoblast proliferation through the Wnt/β-catenin signaling pathway in mice. Bone. 2020;130:115076. 10.1016/j.bone.2019.115076 31622775

[17] Wu Z, Ou L, Wang C, Yang L, Wang P, Liu H, et al. Icaritin induces MC3T3-E1 subclone14 cell differentiation through estrogen receptor-mediated ERK1/2 and p38 signaling activation. Biomed Pharmacother. 2017;94:1–9. 10.1016/j.biopha.2017.07.071 28742995

[18] Guo TF, Zhou MW, Li SH, Ye BL, Chen W, Fu ZB. Long non-coding RNA for metabolism of bone tissue. China J Orthop Traumatol. 2018;31(3):286–91. 10.3969/j.issn.1003-0034.2018.03.020 29600685

[19] Huang Y, Zheng Y, Jia L, Li W. Long noncoding RNA H19 promotes osteoblast differentiation via TGF-β1/Smad3/HDAC signaling pathway by deriving miR-675. Stem Cell. 2015;33(12):3481–92. 10.1002/stem.2225 26417995

[20] He Q, Yang S, Gu X, Li M, Wang C, Wei F. Long noncoding RNA TUG1 facilitates osteogenic differentiation of periodontal ligament stem cells via interacting with Lin28A. Cell Death Dis. 2018;9(5):455. 10.1038/s41419-018-0484-2 PMC5908786 29674645

[21] Gao Y, Xiao F, Wang C, Wang C, Cui P, Zhang X, et al. Long noncoding RNA MALAT1 promotes osterix expression to regulate osteogenic differentiation by targeting miRNA-143 in human bone marrow-derived mesenchymal stem cells. J Cell Biochem. 2018;119(8):6986–96. 10.1002/jcb.26907 29741283

[22] Eckner R, Ewen ME, Newsome D, Gerdes M, Decaprio JA, Lawrence JB, et al. Molecular cloning and functional analysis of the adenovirus E1A-associated 300-kD protein (p300) reveals a protein with properties of a transcriptional adaptor. Genes Dev. 1994;8(8):869–84. 10.1101/gad.8.8.869 7523245

[23] Chen Z-P, Wei J-C, Wang Q, Yang P, Li W-L, He F, et al. Long non‑coding RNA 00152 functions as a competing endogenous RNA to regulate NRP1 expression by sponging with miRNA-206 in colorectal cancer. Int J Oncol. 2018;53(3):1227–36. 10.3892/ijo.2018.4451 29956750

[24] Li R, Xiao C, Liu H, Huang Y, Dilger JP, Lin J. Effects of local anesthetics on breast cancer cell viability and migration. BMC Cancer. 2018;18(1):666. 10.1186/s12885-018-4576-2 PMC6006780 29914426

[25] Liu H, Xiong Y, Zhu X, Gao H, Yin S, Wang J, et al. Icariin improves osteoporosis, inhibits the expression of PPARgamma, C/EBPalpha, FABP4 mRNA, N1ICD and jagged1 proteins, and increases Notch2 mRNA in ovariectomized rats. Exp Ther Med. 2017;13(4):1360–8. 10.3892/etm.2017.4128 PMC5377361 28413478

[26] Clements BA, Incani V, Kucharski C, Lavasanifar A, Ritchie B, Uludağ H. A comparative evaluation of poly-l-lysine-palmitic acid and Lipofectamine™ 2000 for plasmid delivery to bone marrow stromal cells. Biomaterials. 2007;28(31):4693–704. 10.1016/j.biomaterials.2007.07.023 17686514

[27] Liu H, Dilger JP, Lin J. The role of transient receptor potential melastatin 7 (TRPM7) in cell viability: a potential target to suppress breast cancer cell cycle. Cancers. 2020;12(1):131. 10.3390/cancers12010131 PMC7016641 31947967

[28] Chen G, Wang C, Wang J, Yin S, Gao H, Xiang L, et al. Antiosteoporotic effect of icariin in ovariectomized rats is mediated via the Wnt/beta-catenin pathway. Exp Ther Med. 2016;12(1):279–87. 10.3892/etm.2016.3333 PMC4906828 27347050

[29] Ma Y, Chen Y, Lin C, Hu G. Biological functions and clinical significance of the newly identified long non-coding RNA RP1‑85F18.6 in colorectal cancer. Oncol Rep. 2018;40(5):2648–58. 10.3892/or.2018.6694 PMC6151894 30226619

[30] Ying H, Xiao Z-XJ. Targeting retinoblastoma protein for degradation by proteasomes. Cell Cycle. 2006;5(5):506–8. 10.4161/cc.5.5.2515 16552188

[31] Marine JC, Lozano G. Mdm2-mediated ubiquitylation: p53 and beyond. Cell Death Differ. 2010;17(1):93–102. 10.1038/cdd.2009.68 19498444

[32] Keenan SM, Lents NH, Baldassare JJ. Expression of cyclin E renders cyclin D-CDK4 dispensable for inactivation of the retinoblastoma tumor suppressor protein, activation of E2F, and G1-S phase progression. J Biol Chem. 2004;279(7):5387–96. 10.1074/jbc.M310383200 14645251

[33] Meng X, Zhu Y, Tao L, Zhao S, Qiu S. miR-590-3p mediates melatonin-induced cell apoptosis by targeting septin 7 in the human osteoblast cell line hFOB 1.19. Mol Med Rep. 2018;17(5):7202–8. 10.3892/mmr.2018.8729 PMC5928678 29568931

[34] Ma Y, Chen Y, Lin C, Hu G. Biological functions and clinical significance of the newly identified long non‑coding RNA RP1‑85F18.6 in colorectal cancer. Oncol Rep. 2018;40(5):2648–58. 10.3892/or.2018.6694 PMC6151894 30226619

[35] Dalton S, Coverdell PD. Linking the cell cycle to cell fate decisions. Trends Cell Biol. 2015;25(10):592–600. 10.1016/j.tcb.2015.07.007 PMC4584407 26410405

[36] Wang H, Chen Y, Zhai N, Chen X, Gan F, Li H, et al. Ochratoxin A-induced apoptosis of IPEC-J2 cells through ROS-mediated mitochondrial permeability transition pore opening pathway. J Agric Food Chem. 2017;65(48):10630–7. 10.1021/acs.jafc.7b04434 29136370

[37] Liu H, Dilger JP, Lin J. Effects of local anesthetics on cancer cells. Pharmacol Ther. 2020;212:107558. 10.1016/j.pharmthera.2020.107558 32343985

